# Speech phoneme and spectral smearing based non-invasive COVID-19 detection

**DOI:** 10.3389/frai.2022.1035805

**Published:** 2023-01-04

**Authors:** Soumya Mishra, Tusar Kanti Dash, Ganapati Panda

**Affiliations:** Department of Electronics and Communication Engineering, C. V. Raman Global University, Bhubaneswar, India

**Keywords:** COVID-19 detection, machine learning, spectral smearing, phoneme analysis, COVID-19

## Abstract

COVID-19 is a deadly viral infection that mainly affects the nasopharyngeal and oropharyngeal cavities before the lung in the human body. Early detection followed by immediate treatment can potentially reduce lung invasion and decrease fatality. Recently, several COVID-19 detections methods have been proposed using cough and breath sounds. However, very little study has been done on the use of phoneme analysis and the smearing of the audio signal in COVID-19 detection. In this paper, this problem has been addressed and the classification of speech samples has been carried out in COVID-19-positive and healthy audio samples. Additionally, the grouping of the phonemes based on reference classification accuracies have been proposed for effectiveness and faster detection of the disease at a primary stage. The Mel and Gammatone Cepstral coefficients and their derivatives are used as the features for five standard machine learning-based classifiers. It is observed that the generalized additive model provides the highest accuracy of 97.22% for the phoneme grouping “/t//r//n//g//l/.” This smearing-based phoneme classification technique can also be used in the future to classify other speech-related disease detections.

## 1. Introduction

COVID-19 was publicly avowed as an epidemic demanding leading nations with medical prowess to develop faster and more accurate testing mechanisms. Flu, cough, exhaustion, asthma, and pneumonia with fatality have been primarily the clinical symptoms of the affected patients (Peng, 2020). To alleviate the dearth of RT-PCR testing sets, medicos and testing centers had to discover alternate options such as Computed Tomography scans (CT scans) for COVID-19 diagnosis of suspected patients. Some improved COVID-19 detection schemes are used such as contrast limited adaptive histogram equalization and local histogram equalization for extracting significant information from raw chest X-ray images (Narlı, 2021; Narli and Altan, 2022). The velcro-like lung sounds and lung ultrasound readings are also used for the successful detection of COVID-19 (Kiamanesh et al., 2020; Pancaldi et al., 2022). Radiologists have been found to be heavily engaged during the epidemic of COVID-19. They somehow lacked the capacity to decipher a variety of CT scans in due time (Afshar et al., 2021). In addition, clinicians could not as well distinguish COVID-19 from CT scans in remote villages, such as rural regions, because this disease is relatively recent. The importance of reducing the dose of radiation in radiological studies, particularly concerning CT, had become a point of apprehension based on its numerous and dependable medical applications across the globe.

Corona Virus has been primarily hosted on the intra-nasal, bronchial, and lung systems of the human body (Gallo, 2021), and therefore, audio analysis of speech segments from infected samples could potentially indicate respiratory, articulatory, and breathing aberrations as compared with healthy speech samples. Speech-based audio detection of COVID-19 would not only be non-invasive and cost-friendly but can be performed with huge flexibility and portability from any location, adhering to social distancing norms. Speech-based disease recognition has gained immense admiration in recent times predominantly in diagnosing neurodegenerative diseases affecting regular speech patterns. Audio features are explicitly extracted from the concerned database samples, assigned markers for classification, and fed into the system model for training followed by validation and performing an accuracy check (Sharma G. et al., 2020). Phoneme-based disease classification has showcased progressive accuracy with minimum latency in diagnosing several diseases such as stroke, amyotrophic lateral sclerosis (ALS), Parkinson's disease (PD), cleft lip and palate (CLP), primary progressive aphasia, spasmodic dysphonia, Alzheimer's disease, and dementia.

The conventional speech features considered are high-frequency local field potential, zero crossing rate, mean and standard deviation, spikes in the audio signal, Mel-frequency Cepstral coefficients (MFCC), Jitter, shimmer, and voice breaks (Zhang and Wu, 2020). Perceptual linear prediction (PLP), relative spectra (RASTA), and linear prediction coefficients (LPC) have also been reported as instrumental in classification (Moro-Velazquez et al., 2019). Prospective artificial intelligence/machine learning and deep-learning phoneme classification methodologies have been the topic of interest in research advancements for decades (Lamba et al., 2021). Phonemes in the process of articulation can be distinctively segregated into six categories such as stop, affricate, fricative, nasal, and lateral. Subsequently, they can be sub-categorized to the next level of distinction based on modes of sound articulation originating in the vocal tract forming a tubal resonance effect while producing speech (Katamba, 1989). Phonemes, irrespective of dialects, spoken language, or vocabulary adhered across diversities, can alone suffice to be a powerful speech segment for processing speech-based recognition applications. Researchers have actively formulated words made up of relevant phonemes to trigger the appropriate vocal parametric articulations for detecting speech disorders, indicating anomalies (Wielgat, 2008).

### 1.1. Motivation

In previous research outcomes, it has been apparent that variations in phoneme lengths and frequency, as well as changes in phoneme-dependent tone and formant gradients, represent the phonemic segment reliance on phonation and articulation shifts with Parkinson's severity. Yet, there has been a preliminary study on speech-based COVID-19 detection focusing mainly on cough, breath, and vowels (Han et al., 2021; Kumar and Alphonse, 2021) and a generalized comparison of the COVID-19 assessment of phoneme-vowel categories (Boothroyd et al., 1996). Not every affected patient might show cough and shortness of breath as potential symptoms. In this case, phonemes may emerge as worthy indicators for early detection of the disease. The best bet to utilize phonemes as an efficient classification strategy is based on the fact that a speaker need not necessarily generate his samples to train all words in the vocabulary list but only the phonetic segments need to be processed.

### 1.2. Research objective

An effort is initiated in this article to classify COVID-19-affected positive and healthy candidates by disintegrating the audio speech sentence spoken by the concerned specimen into relevantly available English phonemes. The various phonemes are then labeled as positive and healthy classes as demarcated in the referred corpus. In an attempt to enhance classification accuracy, the individual phoneme audio wave has been smeared using low-pass filter noise. Most importantly, the phonemes acquiring the highest classification performance have been concatenated to propose a phoneme group called “buzzword.” The so-called buzzword may be used in the future to detect the disease, evading the dependency on cough or breath samples. In this article, 16 distinct English phonemes with three vowels have been utilized on the available datasets, using 78 feature-sets comprising MFCC, GTCC, and its variant features with five machine-learning classification techniques. The findings of the investigation are as follows:

- Selection of appropriate smearing bandwidth for improving the classification accuracy for different feature sets.
- Use of smearing signal for enhancing the classification accuracy.
- Application of Phoneme-based Buzzwords to assist clinicians and patients with more precise and focused detection mechanism.

## 2. Materials and methods

### 2.1. Dataset

The proposed non-invasive COVID-19 detection scheme is trained and tested in a combined speech dataset, which is prepared from speech samples collected from the Telephone band speech dataset (Ritwik et al., 2020) and Coswara dataset (Sharma N. et al., 2020). A total of 19 speakers' voice has been used in the Telephone band speech dataset, out of which 10 are COVID-19 positive and 9 are healthy. The original speech samples are recorded with 44.1 kHz sampling frequency. But it has been observed that most of the relevant speech components are present within the frequency range of 300 Hz to 3.4 kHz (Jax and Vary, 2004). In the next step, the filtered speech samples are segmented into different phoneme categories using the Audacity Toolkit^1^. There are a total of 432 speech samples in 16 phoneme categories and the details are mentioned in Table 1. From the Coswara dataset, three vowel sounds are taken and the samples are down sampled to 8 kHz sampling frequency. The speech samples are combined and labeled into 19 phoneme categories belonging to vowels, diphthongs, stops, fricatives, glides, liquids, approximants, and nasals. To deal with the insufficient speech samples, the existing speech phoneme samples are processed by an audio data augmentation scheme (Salamon and Bello, 2017). The details of the prepared dataset are listed in Table 1.

**Table 1:** Phoneme database prepared for this study.

**Sl**.	**Phoneme**	**Phoneme category**	**No of speech samples (C-19 p +n)**
1	/b/	Stop	112+104
2	/d/	Stop	108+110
3	/v/	Fricative	108+110
4	/m/	Nasal	108+108
5	/l/	Alveolar Lateral approximant	104+104
6	/f/	Fricative	112+110
7	/Oy/	Diphthong vowel	105+108
8	/r/	Post-alveolar fricative/voiced approximant liquid	108+110
9	/w/	Labio-velar approximant	110+110
10	/p/	Stop	112+112
11	/n/	Nasal	105+104
12	/s/	Fricative	110+110
13	/t/	Stop	112+112
14	/k/	Stop	108+110
15	/h/	Voiceless glottal fricative/Approximants	110+108
16	/g/	Stop	108+110
17	/a/	Vowel	100+100
18	/e/	Vowel	100+100
19	/o/	Vowel	100+100

*(C-19 p +n) denotes (COVID-19 Positive + Healthy).

### 2.2. Proposed methodology

**Figure 1:**
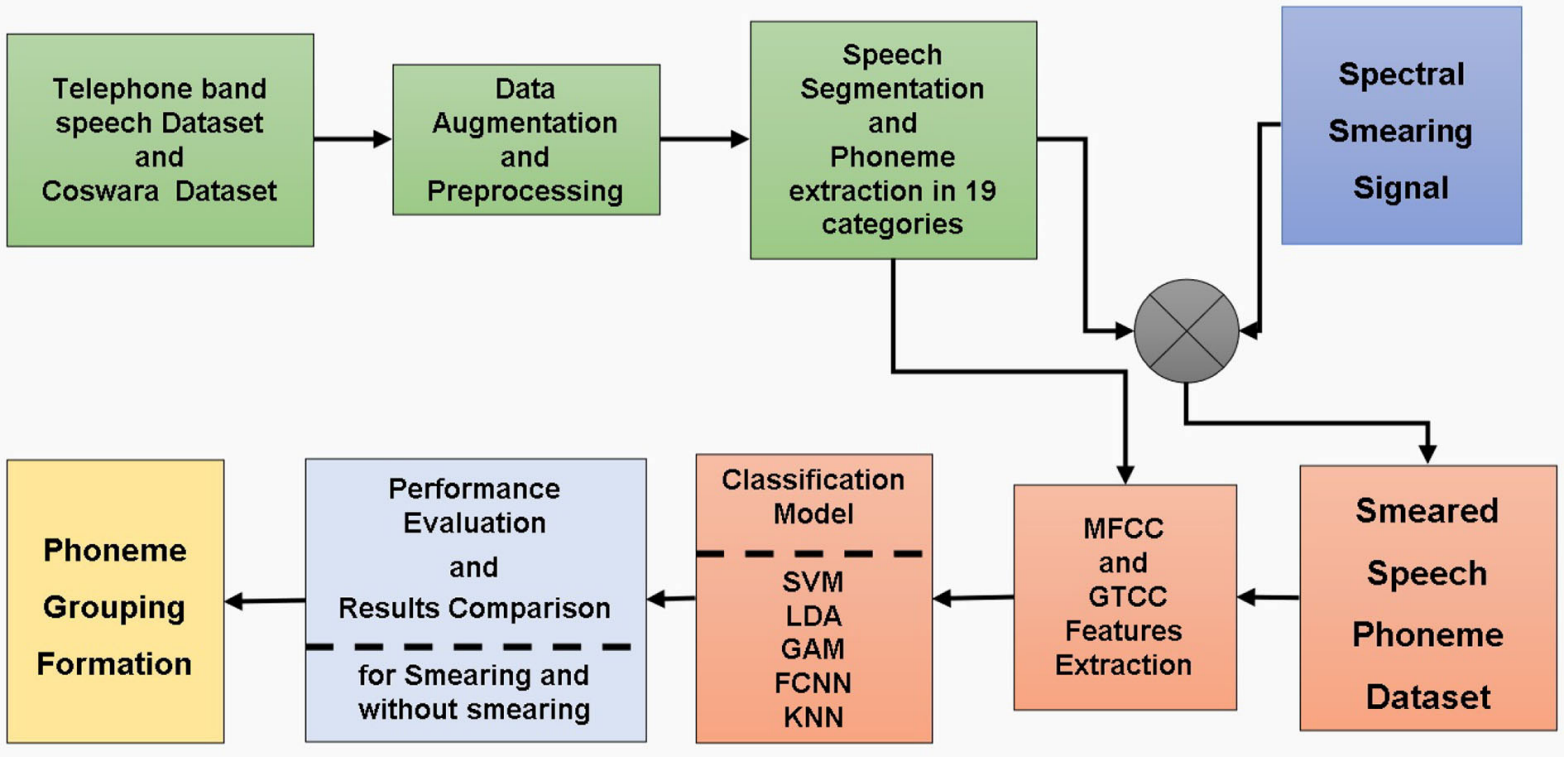
Block diagram of the proposed model.

The proposed method is implemented in the following steps: dataset preparation, spectral smearing, extraction of cepstral features, and training and testing of the classification model. The proposed COVID-19 detection scheme is shown in Figure 1.

### 2.3. Smearing of phonemes

It has been observed that various speech components respond differently to spectral and temporal cues which can be helpful in speech recognition (Xu et al., 2005). The process of spectral smearing is obtained by multiplying the signal with a low-pass filter noise. The approach is known to replace the individual tone factor of the audio-spectrum with a noise band whose center-frequency collides with the particular tone. By this, the bandwidth of the modulated tone is increased twice the tone factor. It has been reported that the effect of smearing has enhanced phoneme detection accuracy (Boothroyd et al., 1996). In Golestani et al. (2009), the authors have conducted experiments on native-language detection to emphasize that certain words can be more conveniently detected at a particular noise configuration than others. This has been accounted for the differential-phoneme-recognition outcomes in a noisy environment. In this case, the speech signals have been smeared using varying SNR levels and it has been observed that this technique provides superior performance as compared to the phonemes without smearing. In yet another study (Shannon, 2005), speech detection has been shown to be possible with highly distorted and degraded audio signals. The spectral information can be modified by smearing to a considerable level till it starts degrading the classification outcome. A study by Goldsworthy (Goldsworthy et al., 2013) has demonstrated evaluating psycho-acoustic phoneme-based identification methods in normal hearing vs. cochlear-implant subjects. The presence of fluctuating noise-makers has shown better interpretation for normal hearing participants. By varying the range of low pass cut-off frequencies, vowel, and consonant recognition scores have shown marked differences illustrating the relativity of spectral resolution (Xu et al., 2005).

In the present study, an attempt has been made to apply spectral smearing to increase phoneme recognition without affecting signal perception by the addition of noise. In the first step, the smearing signal is generated by combining a sinusoidal signal with varied center frequencies and additive white Gaussian noise. This signal is passed through low-pass filters having cut-off frequencies ranging from 10 Hz to 10 kHz. The smearing signal is then multiplied by the phoneme signal to generate the smeared phoneme. The best values of these center frequencies and cut-off frequencies of low-pass filters are calculated based on the classification accuracies from the support vector machine-based classifier. The corresponding values are listed in Tables 2, 3.

**Table 2:** Values of center frequency of sinusoidal signal and cut-off frequencies of low-pass filter for before and after tuning SVM.

**Phonemes**	**Cf/LPBW pre-tuning**	**Accuracy pre-tuning**	**Cf/LPBW post tuning**	**Kernel function/ gamma/C**	**Accuracy post tuning**
STOPS /b/	6.3/6.2	64.25	1.4/4.9	Quadratic /1/ 8.73	89.2
NASALS /m/	4.2/7.9	76.85	1.1/4.3	Gaussian /3.63/238.6	84.2
DIPHTONGS /Oy/	3.1/6	82.6	1.9/2	Gaussian /0.007 /635	84.3
GLIDES + /r/	8.6/8.5	67.7	2.1/4.8	Gaussian/0.001/6523.4	89.9
FRICATIVES /s/	5.6/4.4	67.7	9.9/2.9	Quadratic/1/0.1	85.3
Vowel a	9/7.1	63.6	8/6.6	linear/1/12.6013	79.4

*Cf and LPBW denote Cosine frequency Low Pass Bandwidth in kHz, GLIDES+ denotes the GLIDES, APPROXIMATES, and LIQUIDS.

**Table 3:** Best values of the center frequency of the sinusoidal signal and cut-off frequencies of low-pass filter for the smearing of different phonemes.

**Phonemes**	**Center frequency (kHz)**	**Low-pass filter cut-off frequency (kHz)**
/b/	1.4	4.9
/d/	3.2	1
/v/	8.2	1.6
/m/	1.1	4.3
/l/	4.3	9.6
/f/	6.3	2
/Oy/	1.9	2
/r/	2.1	4.8
/w/	3.1	2.9
/p/	3.7	9.9
/n/	4.4	2
/s/	9.9	2.9
/t/	8.1	4.7
/k/	8.4	9.6
/h/	8.2	4.2
/g/	0.3	6.8
Vowel /a/	8	6.6
Vowel /e/	9.1	4.5

### 2.4. Feature extraction

The objective of signifying an audio signal through its features is primarily to represent a huge data set through a compact form without compromising its vital information. The cepstral features are one of the effective features that are widely used in speech signal processing and mechanical engineering. These features are specially designed by considering the perceptual quality of the human hearing system (Dash et al., 2021a). The following steps are usually performed in cepstral feature extraction:

- Short-time Fourier transforms of windowed speech frames of the input signals.
- Calculation of the short-time energy of speech frame.
- Application of auditory filter bank on the power spectrum.
- Calculation of logarithm and Discrete cosine transform.
- Extraction of specific cepstral features based on the auditory filter bank used.

The third step is the crucial step that works on the conversion between the linear frequency scale and to perceptual frequency scale. Depending on the conversion, two cepstral features such as Mel and Gammatone cepstral Features are used in the proposed implementation scheme. The conversion scale of the Mel scale is mentioned in Equation (1)


(1) $$\begin{array}{c} \begin{array}{l} f_{mel}=2595\;\times \;\log_{10}\;\left(1+\frac{f_{lin}}{700}\right)\\ f_{lin}=700\;\times \left(10^{\left(\frac{f_{mel}}{2595}\right)}-1\right) \end{array} \end{array}$$

Where, *f*_*l*_and *f*_*m*_are the linear scale and mel scale frequencies, respectively.

#### 2.4.1. Mel-scale cepstral features

Studies have shown that short-time speech-based Mel-Cepstral features have been noise evasive, and have significantly detected the pathologies on the vocal tract and vocal folds in past years. The MFCC feature considers human hearing by warping the frequency onto the Mel scale (Milner, 2002). It computes the cepstrum to separate the glottal source and vocal tract filtering information (Quatieri, 2002). The MFCCs have been chosen for this study because, in the presence of voice issues, these have the inherent ability to reflect either irregular movements of the vocal folds or a lack of closures produced by an increase in size or a variation in the attributes of the tissue covering the vocal folds. In this study, 13 feature-based MFCC coefficients, 13 MFCC Delta coefficients, and 13 MFCC Delta-Delta coefficients have been extracted. The delta values represent the first and second derivatives that depict the dynamics of variation in MFCC feature values.

#### 2.4.2. Gammatone cepstral features

Gammatone Cepstral coefficients (GTCCs) are physiologically inspired adaptations that use Gammatone filters and have comparable rectangular bandwidth bands. Several papers (Cheng et al., 2005; Lee et al., 2014) have examined the benefits and use of the Gammatone function in the modeling of the human auditory filter response. The Gammatone filter impulse response is calculated by multiplying a Gamma distribution function by a pure sine wave tone. The delta and double delta GTCC variants (Cheng et al., 2005) are also taken into consideration. In essence, 13 feature-based GTCC coefficients, 13 GTCC Delta coefficients, and 13 GTCC Delta-Delta Coefficients.

### 2.5. Classification

Machine learning-based (ML) classifiers working along with time and frequency extracted features have made substantial progress in this field. Even in noisy conditions, this combination exhibited outstanding accuracies for discrete sound categorization (Dash et al., 2021b). To initiate classification, all the above-mentioned 78 features were extracted from the speech signal and were provided as inputs to the following classifiers. The smeared phonemes were Short-Time Fourier transformed (STFT) using the hamming window of a length of 1,024, having a 30 ms analysis window with a 20 ms overlap. As the noise level varies during the time of recording of different speech samples, the speech enhancement algorithms are widely used to reduce the interfering noise. In the proposed implementation, one of the popular speech enhancement algorithms called the multi-band spectral subtraction method is used in the preprocessing stage before feature extraction (Kamath and Loizou, 2002).

#### 2.5.1. Support vector machines

**Figure 2:**
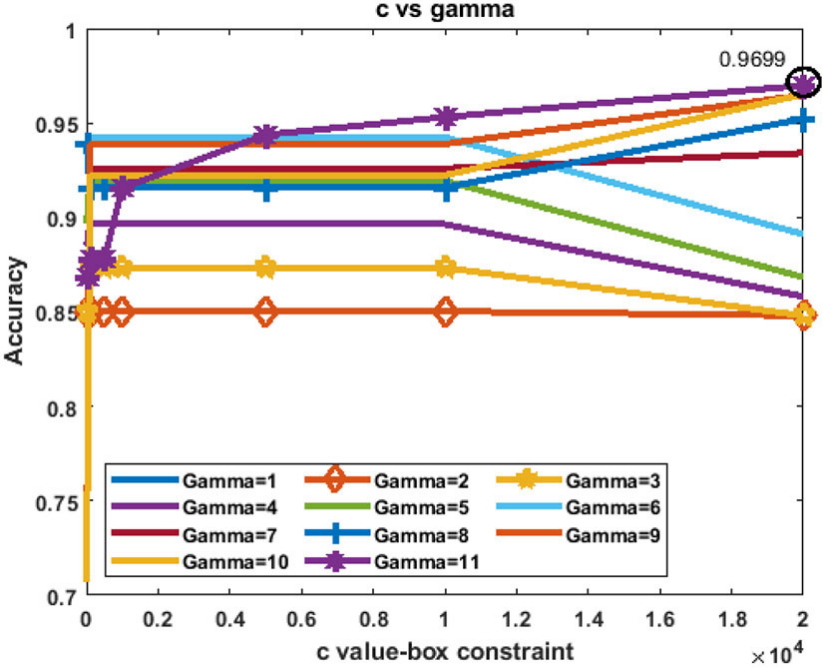
Selection of the values of C and gamma in the SVM classifier.

The primary objective of a support vector machine (SVM) classifier is to obtain the most feasible hyperplanes to assess a proposed model for classification (Soumaya et al., 2021). SVMs have been widely used in speech classification tasks and have shown superior performance (Dash and Solanki, 2019). In this study, bayesian optimization has been applied to select the best SVM parameters. The best values of c and gamma are taken from the comparative analyses between the values of c and gamma vs. classification accuracy as plotted in Figure 2 for the “rbf” kernel.

#### 2.5.2. Linear discriminant analysis

Linear discriminant analysis (LDA) has been employed in multiple speech disease detection or health anomalies through audio analysis (Fredouille et al., 2009; Akbari and Arjmandi, 2014). Fisher's approach is commonly used in linear discriminant analysis. This approach is based on the sample averages and covariance matrices generated from the several groupings that comprise the training sample. Based on the training sample, a discriminant rule is developed and used to classify fresh occurrences into one of the categories. Fisher's linear discriminant analysis is a basic and widely used discriminating approach (Croux et al., 2008).

#### 2.5.3. Generalized additive model

For analyzing the data set and picturing the affiliation of a dependent variable with an independent variable, the generalized additive model (GAM) is used, which evolves from a class of generalized linear models (GLM) (Liu, 2008). Previous studies have shown that the GLM classifier has given appreciable results in temporal feature integration based on music genre classification (Meng et al., 2007). In this case, the boosted tree is used as a shape function for each predictor to capture a nonlinear relation between a predictor and the response variable.

#### 2.5.4. Feed-forward fully connected neural network

Neural network-based classifier models are widely used in speech processing for improved performance (Lopez-Moreno et al., 2016; Dash et al., 2020). In this case, feed-forward fully connected neural network (FCNN) is used with the input layer connected to a fully connected layer of 10 neurons, a ReLU function, followed by a second fully connected layer, a softmax function. A memory-limited device based loss function minimization approach used here is the Broyden-Flecter-Goldfarb-Shanno quasi-Newton algorithm (LBFGS) (Nocedal and Wright, 2006; Hui et al., 2019), where the cross-entropy loss is reduced during the training phase.

#### 2.5.5. K-nearest neighbor

K-nearest neighbor (KNN) is one of the effective and popular classifiers that are used for speech-based applications (Alsmadi and Kahya, 2008). The categorization process is divided into two stages: the first is determining the closest neighbors, and the second is determining the class based on those neighbors. The K-nearest neighbors are selected using the Grid search method that provides the best value of k as 5.

### 2.6. Validation

K-fold cross-validation is a commonly applied validation approach (He et al., 2018). The entire set of voice samples is randomly divided into k equal-sized subgroups. Each fold has an equal proportion of two different types of class labels (glottal and normal stop speech). One of the subsamples is engaged for testing, while the remaining k-1 subsamples can be utilized for training (Altan, 2021, 2022). The process is replayed k times (the folds), for each of the k subsamples serving as testing data. The classification accuracy is calculated for each operation. The mean classification accuracies are calculated using 10 times in 10-fold cross-validation (Muthusamy et al., 2015) for this study. The validation accuracy is computed from confusion metrics as shown below


(2) $$\begin{array}{l} \begin{array}{c} ClassificationAccuracy=\left(\frac{T_{P}+T_{N}}{T_{P}+T_{N}+F_{P}+F_{N}}\right) \end{array} \end{array}$$

where *T*_*P*_ stands for True-Positives, *T*_*N*_ stands for True-Negatives, *F*_*P*_ for False-Positives, and *F*_*N*_ for False- Negatives. The Precision and Recall are calculated as mentioned below.


(3) $$\begin{array}{c} \begin{array}{l} Precision=\frac{T_{P}}{T_{P}+F_{P}}\\ Recall=\frac{T_{P}}{T_{P}+F_{N}}\; \end{array} \end{array}$$

The F-2 score is calculated as


(4) $$\begin{array}{c} \begin{array}{l} \begin{array}{l} F2-Measure=\frac{\left(5\;\times Precision\;\times \;Recall\right)}{\left(4\;\times Precision\;+\;Recall\right)}\\ \; \end{array}\\ \;=\frac{T_{P}}{T_{P}+0.2F_{P}+0.8F_{N}} \end{array} \end{array}$$

The F-2 score is one of the important parameters in medical diagnosis since it indicates the cases who are False Negative (who have COVID-19 infection but have been incorrectly classified as healthy by the model).

## 3. Results and discussions

After completing the experimental setup, the simulations study has been performed on the MATLAB platform using a Core i5, 12GB RAM processor. The results are analyzed in three broad categories: the selection of the best classification model, the effect of smearing, and the formation of the grouping of phonemes.

### 3.1. Performance comparison of different classifiers on smeared phoneme detection

For the selection of the best performing classifier for COVID-19 detection using phoneme and smearing, the performance of the five different classifiers (SVM, LDA, GAM, FCNN, and k-NN) are compared. For this, the classification accuracy, area under the curve (AUC), precision, recall, and F-2 score are used and the results are plotted in Table 4. The average classification performances are listed for six broad categories of phonemes including stops, fricatives, nasals, vowels, voiced, and dipthongs.

**Table 4:** Performance comparison of classifiers on different phoneme categories.

**Smeared phoneme category**	**Model**	**Accuracy**	**AUC**	**Precision**	**Recall**	**F-2 Score**
STOPS	SVM	0.9 ± 0.0045	0.87	0.92 ± 0.0012	0.9 ± 0.004	0.75 ± 0.002
/b/,/d/,	LDA	0.81 ± 0.025	0.83	0.81 ± 0.007	0.86 ± 0.0063	0.70 ±0.0069
/g/,/k/,	GAM	0.9± 0.02	0.96	0.9 ± 0.0033	0.9 ± 0.0047	0.75 ± 0.0033
/t/,/p/	FCNN	0.85 ±0.016	0.89	0.87 ± 0.0022	0.86 ± 0.0033	0.72 ± 0.0022
	KNN	0.80± 0.023	0.79	0.86 ± 0.0067	0.77 ± 0.0031	0.65 ± 0.0071
FRICAT	SVM	0.92± 0.01	0.8	0.97 ± 0.0032	0.92 ± 0.0058	0.69 ± 0.0041
IVES	LDA	0.72 ±0.02	0.74	0.67 ± 0.0015	0.70 ± 0.0033	0.57 ± 0.0011
/f/,/s/,/v/	GAM	0.89± 0.2	0.94	0.89 ± 0.0073	0.92 ± 0.0064	0.76 ± 0.0022
	FCNN	0.64 ±0.04	0.59	0.70 ± 0.0017	0.68 ± 0.001	0.57 ± 0.0046
	KNN	0.82± 0.015	0.77	0.84 ± 0.0069	0.82 ± 0.0022	0.69 ± 0.004
NASALS	SVM	0.87 ±0.02	0.88	0.87 ± 0.0033	0.87 ± 0.0071	0.73 ± 0.006
/m/,/n/	LDA	0.67 ± 0.06	0.68	0.70 ± 0.004	0.63 ± 0.0022	0.53 ± 0.0011
	GAM	0.94 ± 0.01	0.98	0.95 ± 0.001	0.93 ± 0.0023	0.77 ± 0.0066
	FCNN	0.87± 0.01	0.91	0.77 ± 0.0014	0.89 ± 0.008	0.72 ± 0.004
	KNN	0.77 ±0.02	0.76	0.78 ± 0.0012	0.76 ± 0.0011	0.63 ± 0.0032
VOWELS	SVM	0.78 ± 0.0012	0.77	0.78 ± 0.0046	0.78 ± 0.0010	0.70 ± 0.0067
/a/,/e/, /o/	LDA	0.63 ± 0.0071	0.68	0.59 ± 0.0012	0.73 ± 0.0012	0.58 ± 0.0033
	GAM	0.84 ± 0.0045	0.91	0.79 ± 0.0033	0.85 ± 0.004	0.69 ± 0.0012
	FCNN	0.85 ± 0.0023	0.90	0.89 ± 0.0047	0.83 ± 0.0033	0.69 ± 0.001
	KNN	0.64 ± 0.0017	0.64	0.55 ± 0.0014	0.68 ± 0.004	0.54 ± 0.0064
GLIDES+,	SVM	0.81 ± 0.006	0.81	0.81 ± 0.0035	0.81 ± 0.0010	0.73 ± 0.0022
/l/ /w/	LDA	0.80 ± 0.0011	0.74	0.75 ± 0.0044	0.86 ± 0.006	0.7 ± 0.004
/r/ /h/	GAM	0.96 ± 0.0014	0.98	0.95 ± 0.008	0.95 ± 0.0010	0.8 ± 0.0041
	FCNN	0.57 ± 0.0079	0.66	0.55 ± 0.0011	0.57 ± 0.001	0.5 ± 0.007
	KNN	0.82 ± 0.0015	0.83	0.85 ± 0.0066	0.79 ± 0.002	0.67 ± 0.006
DIPTHO	SVM	0.79 ± 0.0028	0.76	0.78 ± 0.0044	0.78 ± 0.0035	0.75 ± 0.008
NGS	LDA	0.63 ± 0.0067	0.54	0.68 ± 0.0033	0.54 ± 0.0022	0.47 ± 0.006
/Oy/	GAM	0.87 ± 0.0011	0.93	0.85 ± 0.0014	0.85 ± 0.001	0.71 ± 0.0022
	FCNN	0.88 ± 0.0036	0.80	0.88 ± 0.0026	0.86 ± 0.006	0.72 ± 0.001
	KNN	0.67 ± 0.0044	0.67	0.73 ± 0.007	0.57 ± 0.007	0.5 ± 0.0041

In terms of classification accuracies, /t/, /a/, /f/, /k/, /l/, /m/, /n/, /o/, and /r/ have obtained the best results under GAM Classifier. Similarly, /b/, /e/, /g/, and /oy/ have achieved their highest classification accuracies under FCNN Classifier. LDA Classifier outperformed the rest for /p/, and /v/. SVM offered the highest classification accuracies for both /w/, and /s/. Finally, KNN achieved the best performance in the case of /h/ phoneme. Conclusively, GAM delivers an overall best performance for all phonemes as compared to other classifiers.

### 3.2. Comparison of classification accuracy between non-smeared and smeared phonemes

**Figure 3:**
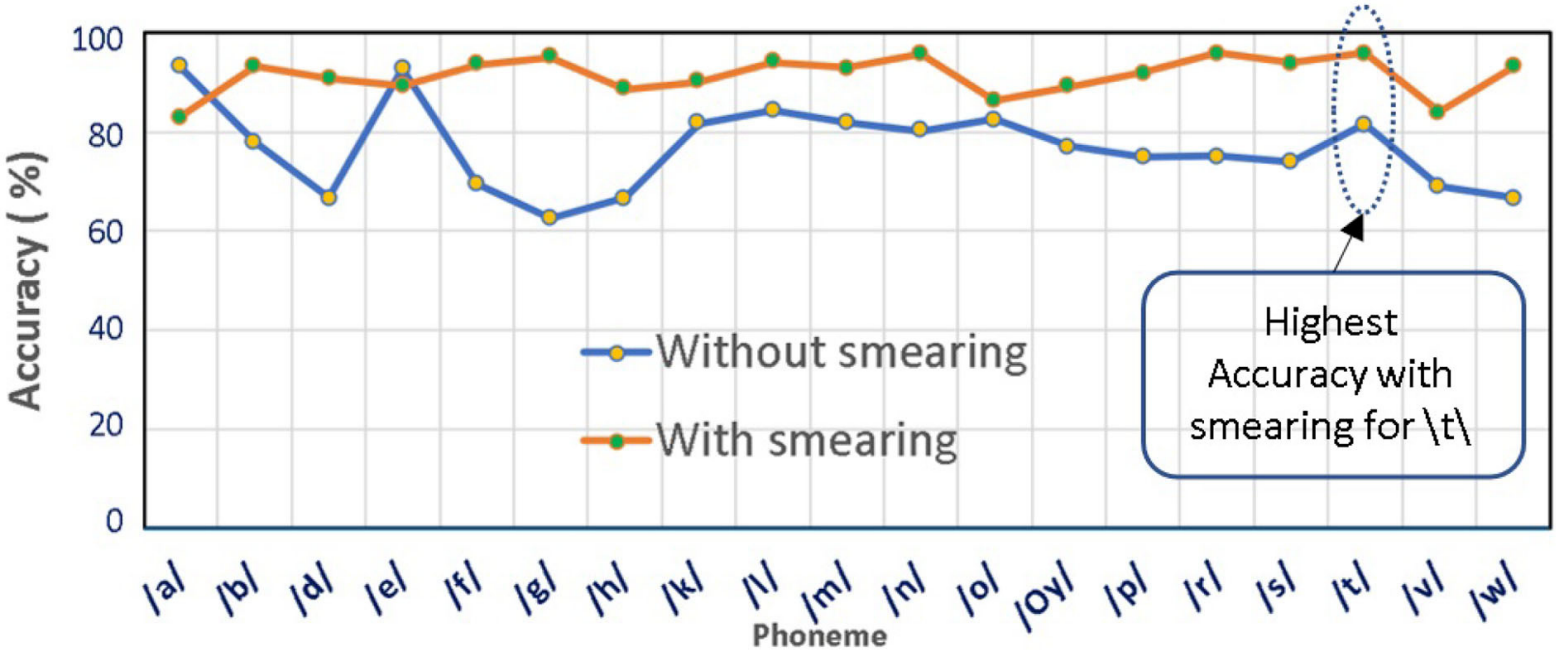
Performance comparison of phonemes in non-smearing and smearing conditions.

To detect the effect of smearing on the classification performance, a comparative analysis is carried out between the phonemes with and without smearing. For the classification of the best performing model from the classification analysis, GAM is used. The same 78-dimensional feature vector sets have been extracted from corresponding phoneme samples. The simulation results are shown in Figure 3.

It is evident from the above figure that the smearing of phonemes yields appreciably better classification accuracies in the majority of the cases. The phoneme /t/ exhibits the highest classification accuracy of 95.92%, and phoneme/a/ exhibits the lowest accuracy of 83.08% under the smeared conditions.

### 3.3. Phoneme groupings

**Figure 4:**
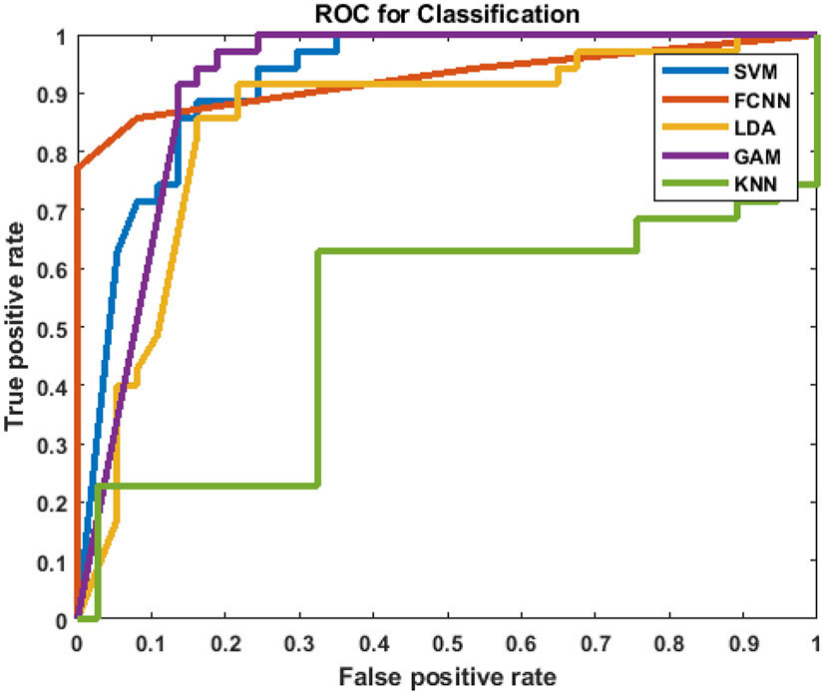
ROC-AUC plot for the phoneme group /t//r//n//g//l/.

**Figure 5:**
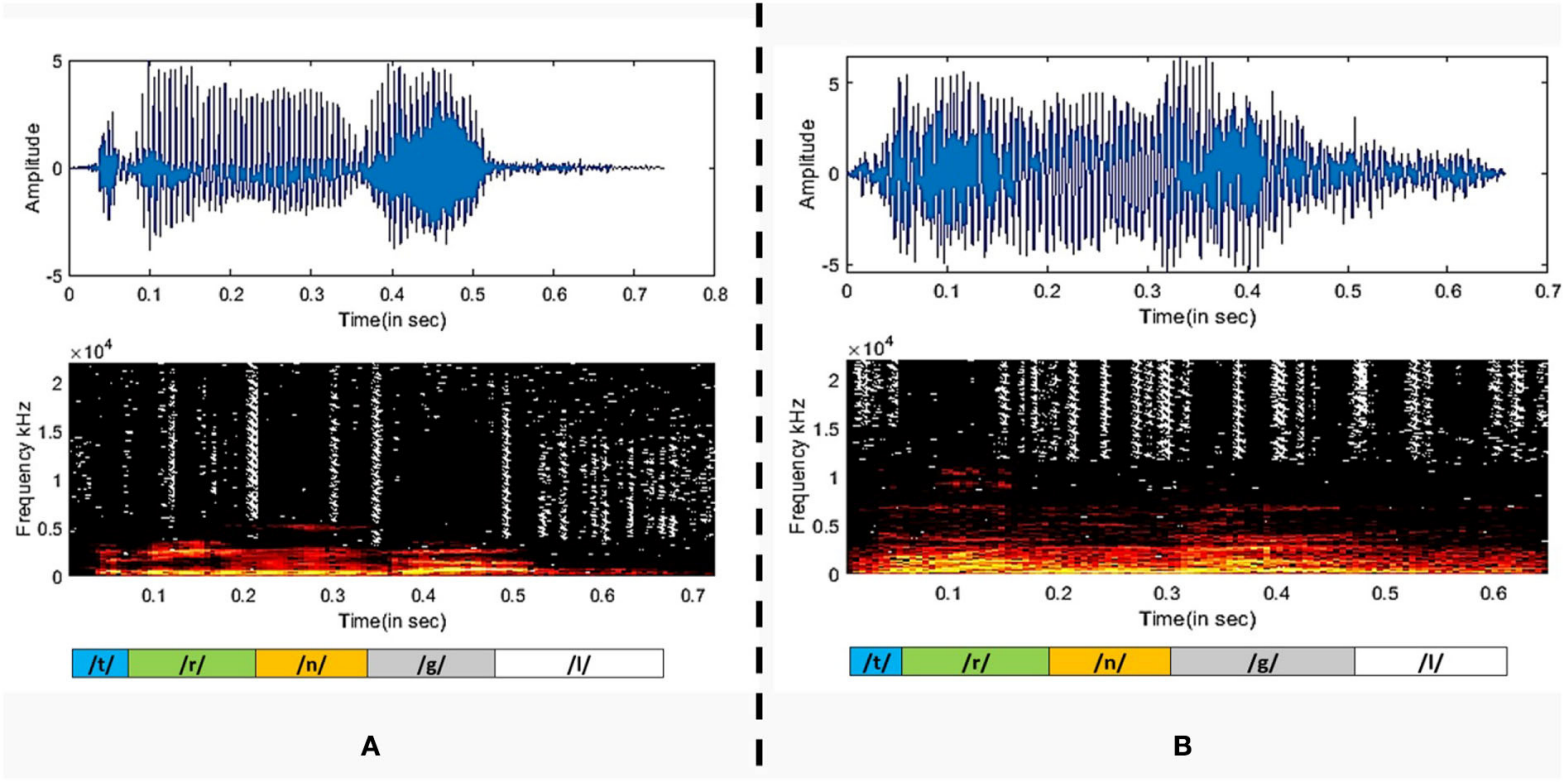
The plot of the Spectrogram of **(A)** COVID-19 positive sample and **(B)** a healthy sample.

After analyzing the classification performance of smearing and individual phonemes, a phoneme grouping based approach is adopted. Based on the individual classification accuracy of phonemes, the 3-tuple and 5-tuple phoneme buzzwords are created by combining the high-performing individual phonemes (Moro-Velazquez et al., 2019). By taking the first reference level of 95.67% classification accuracy, the first phoneme group of “/t/-/r/-/n/” is used as a 3-tuple buzzword. Then, the threshold is set at 94.07% classification accuracy to form the second phoneme group of “/t/-/r/-/n/-/g/-/l/.” The best performing five phonemes are then combined. In these combinations, the phoneme classification accuracies are taken in descending order where the /t/ is having the highest classification accuracy and /l/ is having the lowest classification accuracy among the group. Audacity software is used to combine the individual phonemes to form 104 speech samples in both the categories of COVID-19 positive and healthy for the phoneme group of “/t/-/r/-/n/” and “/t/-/r/-/n/-/g/-/l/.” The same 78-dimensional feature vectors are extracted and applied to the GAM classifier and the results are listed in Table 5. The ROC-AUC curve is plotted for the phoneme group /t//r//n//g//l/ in Figure 4 and the comparison between spectrogram of COVID-19 positive sample and healthy sample is plotted in Figure 5.

**Table 5:** Comparison of GAM Classification performance for best phoneme categories and groupings.

**Phoneme grouping**	**Classification accuracy**	**Precision**	**Recall**	**F-2 Score**
/t/	0.95 ± 0.01	0.95 ± 0.0021	0.93 ± 0.030	0.94 ± 0.014
/r/	0.94 ± 0.01	0.94 ± 0.001	0.94 ± 0.021	0.94 ± 0.017
/n/	0.94 ± 0.012	0.94 ± 0.0024	0.94 ± 0.003	0.94 ± 0.01
/g/	0.93 ± 0.012	0.94 ± 0.013	0.94 ± 0.001	0.93 ± 0.008
/l/	0.92 ± 0.016	0.93 ± 0.015	0.93 ± 0.0012	0.93 ± 0.006
/t//r//n/	0.96 ± 0.0011	0.97 ± 0.001	0.96 ± 0.001	0.96 ± 0.004
/t//r//n//g//l/	0.97 ± 0.0005	0.97 ± 0.001	0.97 ± 0.001	0.97 ± 0.0013


It is observed that the phoneme group with the buzzword “/t//r//n//g//l/” performs better as compared to /t//r//n/. The spectrograms of the buzzword “/t//r//n//g//l/” are plotted for COVID-19 positive and healthy speech samples are plotted in (Narlı, 2021).

**Figure 6:**
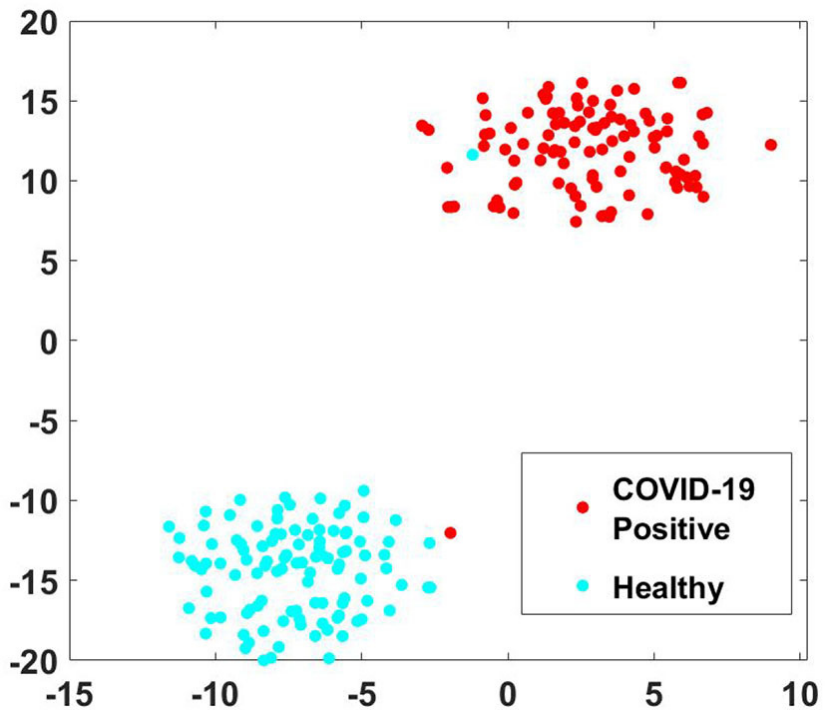
t-SNE plots of phoneme group buzzword “/t//r//n//g//l/” for MFCC and GTCC features.

A person affected by COVID-19 may lack in energy to produce sound, thus disrupting the normal speech production phenomena. In the stage of sound phonation, the sub-glottal thrust must cross a certain threshold to set the vocal folds in vibration. If the respiration stage of speech production is interrupted, the phonation of the larynx will be accordingly compromised (Asiaee et al., 2020). Therefore, the audio waveform of the plosive /t/ in healthy candidate exhibits strong energy compaction due to sufficient sub glottal pressure as compared to the diseased case. The healthy vocal folds exhibit glottal closures with a trail of strong impulses due to the quick closure of vocal folds, whereas a disordered vocal fold produces a weak impulse due to the incomplete closure of vocal folds (Mandal and Rao, 2018). The ability to increase or decrease vocal cord length and tension governs the frequency at which the cord vibrates and, consequently, the pitch of the sound produced. As the mass of the vocal cords increases, the vibrating frequency and pitch decrease (Dettelbach et al., 1994). In the above spectrograms, the healthy waveform depicts equivalent variation for all phonemes, whereas, in the case of COVID-19 affected sample, certain phonemes are subdued as compared to others. To further evaluate the effectiveness of the extracted MFCC and GTCC features for phoneme group buzzword “/t//r//n//g//l/,” the t-SNE plot is shown in Figure 6 (der Maaten and Hinton, 2008). It is observed that in the input space, the pattern of the extracted features is linearly separable which improves the performance of the classification especially the phoneme group buzzword “/t//r//n//g//l/.”

This approach to phoneme grouping has the advantage of designing a low computational complexity based COVID-19 detection model as the individual phonemes are not recorded and the group has a higher classification accuracy as compared to individual phonemes.

## 4. Conclusion

In this study, a hybrid model is designed for the detection of COVID-19 from speech signals by combining phoneme-based signal analysis and spectral smearing. The performance of the detection model is evaluated for 19 individual phonemes and two phoneme groupings using five ML-based classifiers. It is observed that the GAM model performs appreciably better for most pathological phoneme detection. These methods are expected to perform well among suspected COVID-19 patients with minimal or no cough and shortness of breath. Due to insufficient audio samples present in the corpus and to avoid the issues of imbalanced data, the final dataset has been created with the help of data augmentation prior to further processing. In the future, a phone or a web application may be developed for detection based on this buzzword. This proposed methodology needs to be clinically validated in hospitals with large speech datasets.

## Data availability statement

The original contributions presented in the study are included in the article/supplementary material, further inquiries can be directed to the corresponding authors.

## Author contributions

SM formulated the problem statement and simulated the experiment. TD contributed in drafting the manuscript. GP revised and modified the manuscript. All authors contributed to the article and approved the submitted version.

## Footnotes

^1^ http://www.audacityteam.org/
